# The specific metabolome profiling of patients infected by SARS-COV-2 supports the key role of tryptophan-nicotinamide pathway and cytosine metabolism

**DOI:** 10.1038/s41598-020-73966-5

**Published:** 2020-10-08

**Authors:** H. Blasco, C. Bessy, L. Plantier, A. Lefevre, E. Piver, L. Bernard, J. Marlet, K. Stefic, Isabelle Benz-de Bretagne, P. Cannet, H. Lumbu, T. Morel, P. Boulard, C. R. Andres, P. Vourc’h, O. Hérault, A. Guillon, P. Emond

**Affiliations:** 1 UMR 1253, iBrain, équipe « neurogénomique et physiopathologie neuronale », INSERM, Université de Tours, Tours, France; 2 Service de Biochimie et Biologie Moléculaire, CHU de Tours, Tours, France; 3 Service de Pneumologie et d’Explorations Fonctionnelles Respiratoires, CHU, Tours, France; 4 Centre d’Etude Des Pathologies Respiratoires, INSERM U1100, Université de Tours, Tours, France; 5 INSERM U1259, MAVIVH, Université de Tours, Tours, France; 6 Service de Médecine Interne et Maladies Infectieuses, CHU de Tours, Tours, France; 7 Service de Bactériologie-Virologie-Hygiène, CHU de Tours, Tours, France; 8 Department of Biological Hematology, University Hospital of Tours, Tours, France; 9 CNRS ERL 7001 LNOx and EA, University of Tours, 7501 Tours, France; 10 Intensive Care Unit, Research Center for Respiratory, Tours University Hospital, Tours, France; 11 Service de Médecine Nucléaire in vitro, CHRU de Tours, Tours, France

**Keywords:** Metabolomics, Biomarkers, Virology

## Abstract

The biological mechanisms involved in SARS-CoV-2 infection are only partially understood. Thus we explored the plasma metabolome of patients infected with SARS-CoV-2 to search for diagnostic and/or prognostic biomarkers and to improve the knowledge of metabolic disturbance in this infection. We analyzed the plasma metabolome of 55 patients infected with SARS-CoV-2 and 45 controls by LC-HRMS at the time of viral diagnosis (D0). We first evaluated the ability to predict the diagnosis from the metabotype at D0 in an independent population. Next, we assessed the feasibility of predicting the disease evolution at the 7th and 15th day. Plasma metabolome allowed us to generate a discriminant multivariate model to predict the diagnosis of SARS-CoV-2 in an independent population (accuracy > 74%, sensitivity, specificity > 75%). We identified the role of the cytosine and tryptophan-nicotinamide pathways in this discrimination. However, metabolomic exploration modestly explained the disease evolution. Here, we present the first metabolomic study in SARS-CoV-2 patients which showed a high reliable prediction of early diagnosis. We have highlighted the role of the tryptophan-nicotinamide pathway clearly linked to inflammatory signals and microbiota, and the involvement of cytosine, previously described as a coordinator of cell metabolism in SARS-CoV-2. These findings could open new therapeutic perspectives as indirect targets.

## Introduction

COVID-19 due to SARS-CoV-2 viral infection most often presents as mild disease but can develop into life-threatening pneumonia. Patients with COVID-19 pneumonia may progress to respiratory failure, which then typically occurs 7–10 days after the first symptoms^1,2^. Although the overall case fatality rate is estimated to range from 0.25 to 3%, in some countries survival may be as low as 30% in patients admitted to an intensive care unit^3–5^.

As of now, no treatment has been proven to reduce risk of respiratory failure or mortality in COVID-19. Clinical trials of antivirals (remdesivir, lopinavir/ritonavir), repurposed drugs (hydroxychloroquine, interferon beta), and monoclonal antibodies targeting specific pro-inflammatory cytokines (tocilizumab, anakinra) are underway^6^. As no definitive recommendations were published yet, the therapeutically strategies remain to be determined.

The poor understanding of the biological mechanisms underlying COVID-19 hinders developments of evidence-based therapeutic strategies. Although hyperinflammation is understood to play a major role in patients with severe COVID-19^7^, it is unclear to what extent direct cellular lesions, activation of coagulation pathways, or immune responses contribute to the clinical picture. Both clinical (male sex, older age, previous lung or cardiovascular disease, obesity, hypertension) and biological (inflammation, coagulation) markers are associated with poor outcomes in COVID-19. Interestingly, conditions associated with metabolic disturbances, such as obesity or diabetes, are associated with a high risk of severe COVID-19^8,9^.

The strong association between obesity, diabetes and older age with clinical outcomes suggests that metabolic disturbances may play key roles in COVID-19, and deserves to be explored in depth. In this context, metabolomics may be a strategy of choice. Metabolomics is based on the global search for metabolites, defined as small molecules^10^, thus providing a metabolic profile—or “metabotype” that directly reflects the metabolic status of different organs and tissues because of continuous exchanges of metabolites between tissues and fluids. This approach is widely used to identify specific metabolic profiles for diagnosis and prognostication, and may lead to personalized medicine strategies. Beyond patient profiling, metabolic signatures enable to better understand biological pathways associated with disease and generate mechanistic hypotheses. A very recent Chinese work convinced us that proteomic combined to metabolomics may help in the identification of molecular changes in COVID-19 patients during the course of infection evolution^11^.

The general hypothesis of this project was that it is possible to establish a COVID-19 metabolic signature at the time of the diagnosis in a population naïve for anti-viral treatment. The primary aims were thus to characterize early plasma metabolome of French COVD-19 patients in comparison to a matched population and to evaluate the ability to predict the diagnosis in an independent population. Based on the same approach, our second aim was to assess the relation between metabolome profile and clinical characteristics and we evaluated the feasibility to prognosticate clinical evolution of COVID-19 patients. Finally, we highlighted the metabolic pathways involved in the discrimination between diagnosis and prognosis groups.

## Material and methods

### Patients

All adult patients hospitalized in Tours University Hospital for suspected COVID-19 (based on standard clinical criteria mainly including fever, cough, dyspnea and diarrhea) from April 8th to April 20th, 2020 were included. Plasma samples collected for routine biological exploration less than 7 days after diagnosis of SARS CoV-2 infection and with at least 100 µL remaining plasma were used for metabolic exploration. All analytical methods were carried out in accordance with relevant guidelines and regulations. All experimental protocols were approved by the University Hospital of Tours (“cellule de recherches non interventionnelles”). All patients included in this study were informed in writing regarding the collection of their samples remaining from routine biological analyses for research aims and given the right to refuse such uses. In addition, all patients were informed about the data obtained and about their right to access these data, according to articles L.1121-1 and R1121-2 of the French Public Health Code. Participants had to give written refusal about the use of samples and/or clinical data. However, none of them refused, thus considering that informed consent was obtained for each participant enrolled in the study.

Gender, age, Body Mass Index (kg/m^2^), presence of hypertension, cardiovascular risk markers, diabetes, dyslipidemia, smoking history and ongoing treatment were collected. The Charlson index considered as the most widely used comorbidity index^12^ and containing 19 items including diabetes with diabetic complications, congestive heart failure, peripheral vascular disease, chronic pulmonary disease, …, each of which weighted according to their potential influence on mortality, was evaluated for all patients. To describe clinical outcomes between the time of SARS-CoV-2 RTPCR (D0) and the 7th day after sampling (D7) time points, the World Health Organization ordinal scale for clinical improvement (0: uninfected, 1: no limitation of activities, 2: limitation of activities, 3: hospitalized, no oxygen therapy, 4: oxygen by nasal mask of prongs, 5: non-invasive ventilation of high-flow oxygen, 6: intubation and mechanical ventilation, 7: ventilation + additional organ support, 8: death) was used. The evolution of WHO ordinal scale was defined by 3 groups : same, increased or decreased score. Since the WHO ordinal scale could not be used in patients who were discharged from the hospital due to missing data, outcomes at the 15th day after SARS-CoV-2 RTPCR were recorded as death, hospitalization, or hospital discharge.

The following routine biological parameters were also noted at the time of SARS-CoV-2 RTPCR (D0) and 7 days after (D7): ASpartate Aminino Transferase (ASAT), Alanine Amino Transferase (ALAT), creatinine, urea, ferritin, Protein C Reactive (CRP), Creatine Kinase (CK), Lactate DesHydrogenase (LDH), troponin T, lymphocytes count, haemoglobin, platelets counts and D dimer concentrations.

### SARS-CoV-2 polymerase chain reaction

Diagnosis of COVID-19 relied on SARS-CoV-2 RTPCR performed on naso-pharyngeal swabs in transport medium (UTM or Eswab) or broncho-alveolar lavage fluid. Samples were stored at + 4 °C before analysis. SARS-CoV-2 RNA was amplified by real-time RT-PCR targeting RdRp, E and/or N genes, using Allplex 2019-nCOV (Seegene), Abbott RealTime SARS-CoV-2 (Abbott) or Bosphore 2019-nCoV (Anatolia GeneWorks) assays. Patients with positive RTPCR had proven COVID-19 and were allocated to group C +. The other patients who were suspected for SARS-CoV-2 infection but who were negative for the RTPCR test were allocated to group C −. This technique presents a high sensitivity (limit of detection ranging from 25 to 100 copies of RNA per reaction), requiring a sample collection close to COVID-19 infection (before 14 to 21 days).

### Metabolomics analysis

LC-HRMS analysis was performed as previously described^13^ after extraction with 100 µL of methanol from 20 µL of plasma. A UPLC Ultimate WPS-3000 system (Dionex, Germany) coupled to a Q-Exactive mass spectrometer (Thermo Fisher Scientific, Bremen, Germany) and operated in positive (ESI +) and negative (ESI −) electrospray ionization modes (analysis for each ionization mode) was used for this analysis. Liquid chromatography was performed using a Phenomenex Kinetex 1.7 µm XB—C18 column (100 mm × 2.10 mm) maintained at 40 °C. Two mobile phase gradients (preceded by an equilibrium time of 3 min; A: H_2_O + 0.1% formic acid; B: methanol + 0.1% formic acid were used. The gradient was maintained at a flow rate of 0.4 mL/min. The multistep gradient was programmed as follows: 0–2 min, 0.1% B; 2–6 min, 0.1–25% B; 6–10 min, 25 − 80% B; 10 − 12 min, 80–90% B; 12–16.5 min, 90–99.9% B; 16.5–20 min, 99.9–0.1% B.

Two different columns were used to increase the metabolic coverage. Accordingly, a hydrophilic interaction liquid chromatography (HILIC) column (CORTECS UPLC HILIC 150 mm × 2.1 mm × 1.6 µm (WATERS)) was also used. Two mobile phase gradients were also used (A: H_2_O + 10 mM formiate NH4 + 0.5% formic acid; B: acetonitrile + 10 mM formiate NH4 + 0.5% formic acid) and the gradient was maintained at a flow rate of 0.3 mL/min. The multistep gradient was programmed as follows: 0–1.5 min, 95% B; 1.5–8 min, 95–82% B; 8–15 min, 82–75% B; 15–15.5 min, 75–25% B; 15.5–16 min, 25–3% B; 16–22 min, 3–95% B. The volume of injection was 5 µL.

During the full-scan acquisition (full MS, AGC target 1e6, maximum injection time 250 ms), which ranged from 58 to 870 m/z, the instrument operated at 70,000 resolution (m/z = 200).

As required for all biological analyses, pre-analytical and analytical steps of the experiment were validated by findings of Quality Control (QC) samples. Quality control solution was prepared from a mix of 20 random samples from our cohort. Thus this pool was representative of our cohort and they were extracted exactly with the same process as patients’ samples. Consequently, they represented both, technical variation and extraction variation. Coefficients of variation [CV% = (the standard deviation/ mean) × 100], were calculated from all metabolites data. Metabolites with a CV in QCs > 30% were excluded from the final dataset. QCs were analyzed at the beginning of the run, every 10 samples and at the end of the run.

A targeted analysis was applied on the samples, based on a library of standard compounds (Mass Spectroscopy Metabolite Library (MSML) of standards, IROA Technologies). The following criteria were followed to identify the metabolites: (1) retention time of the detected metabolite within ± 20 s of the standard reference, (2) exact measured molecular mass of the metabolite within a range of 10 ppm around the known molecular mass of the reference compound, and (3) correspondence between isotopic ratios of the metabolite and the standard reference. The signal value was calculated using Xcalibur software (Thermo Fisher Scientific, San Jose, CA) by integrating the chromatographic peak area corresponding to the selected metabolite. At this step, the dataset contained the identity of the metabolites and the corresponding area for all the samples after analysis and validation by the specialist of mass spectrometry. Data were normalized to the sum, log-transformed and autoscaled before statistical analysis.

### Statistical analysis

#### Univariate analysis

Comparison of continuous parameters defining population characteristics, such as age, BMI, Charlson index and delay between SARS-Cov-2 RTPCR and sample collection, was done by Student or Wilcoxon test, depending on the distribution’s normality (Shapiro test). WHO ordinal scale at each time point was considered by class defined by the cut off 3 (oxygen request) and was analysed by the Chi^2^ test, as all the qualitative data.

The univariate analysis of metabolites levels between groups was based on fold-change (FC) values and the threshold of significance with the volcano plot and the non-parametric Wilcoxon test using the free software Metaboanalyst, version 4.0 (www.metaboanalyst.ca/faces/home.xhtml). The x-axis represents the fold change between the subject groups (log scale). The y-axis represents the adjusted *p*-value for t-tests of differences between samples (negative log scale). We also used the False Discovery Rate to account for multiple testing and to highlight the most discriminant parameters (Benjamini and Hochberg). We highlighted only metabolites with *p* < 0.1 and FC (C −/C +) > 1.5 or < 0.67. In case of comparison between more than 2 groups, Kruskal Wallis test was performed.

#### Multivariate analysis

Multivariate analysis was performed (1) to evaluate the relation between metabolome and diagnosis as well as clinical evolution within the C + cohort (based on WHO ordinal scale evolution at D7 and the future of patients at D15 i.e. hospital discharge, hospitalization or death), and (2) to test the ability to predict the diagnosis and the clinical evolution within the C + cohort, defined as previously.

Classification was performed by unsupervised Principal Component Analysis (PCA) to evaluate distribution of samples and identify outsiders and supervised analysis based on Partial Least-Squares Discriminant Analysis (PLS-DA). The score plots show the classified samples, the values of Variable Influence on Projection (VIP) represent the importance of the compound (metabolites) for the PLS-DA models. Metaboanalyst modelling includes leave-one-out cross-validation (LOOCV). To test the relevance of these selected compounds, the quality of the model built from them was assessed by prediction accuracy and permutation test. The performance measures of the permutated data usually form a normal distribution, and if the performance score of the original data lies outside the distribution, then the results are considered to be significant.

Then, we established an independent validation for predictions’ models. Thus, we used two independent cohorts (training and test sets) to establish the reliability of our models. We randomly divided our dataset into a training set comprising 80% of the C + and C − participants, and a test set consisting of the remaining 20% of patients (random selection of subjects within each group: C + and C −). Receiver Operating Characteristic (ROC) curves were generated by Monte-Carlo cross validation (MCCV) using balanced sub-sampling. In each MCCV, two thirds (2/3) of the samples were used to evaluate the feature importance. The top 5, 10 …100 (max) important features were then used to build classification models which is validated on the 1/3 the samples that were left out. The procedures were repeated multiple times to calculate the performance and confidence interval of each model. The method for classification and for features ranking was the PLS-DA as previously defined. Thus (1) we generated different PLS-DA models according to variable numbers of features in the training set, (2) we chose the model that provided the highest AUC with less than 10 metabolites to keep a population/metabolite ratio at about 10, (3) and finally we selected the metabolites the most often discriminant in the models to predict the diagnosis or the clinical evolution in the test set. We independently repeated this process 5 times to estimate the sensitivity, specificity, and positive predictive as well as negative predictive values (PPV: positive predictive value, NPV: negative predictive value) of prediction in the test set.

### Pathway analysis

Venn diagrams, aiming at revealing the metabolites most significantly associated with the diagnosis or disease evolution after the different predictions based on PLS-DA models were constructed (free software Venny 2.1, https://bioinfogp.cnb.csic.es/tools/venny/).

Enrichment and pathway analysis were systematically performed from all discriminant metabolites highlighted in the PLS-DA models. Metabolic pathway enrichment analysis and pathway topology analysis was conducted using MetaboAnalyst computational platform (https://www.metaboanalyst.ca/), which computes a single *p* value for each metabolic pathway. Pathway topology analysis applies graph theory to measure a given experimentally identified metabolite’s importance in a pre-defined metabolic pathway. Measurements were computed using centrality to estimate the relative importance of individual nodes to the overall network.

Pathway analysis calculated pathway impact that represents a combination of the centrality and pathway enrichment results; higher impact values represent the relative importance of the pathway, relative to all pathways included in the analysis. The pathway impact value was calculated as the sum of importance measures of the metabolites, normalized by the sum of importance measures of all metabolites in each pathway^14^. Metabolic pathways are represented as a network of chemical compounds with metabolites as nodes and reactions as edges. Major criteria are used to perform an informative analysis regarding the quality of pathway data^15^.

## Results

### Patients

A total of 100 patients were included, of which 45 were C − and 55 were C +. Patients’ characteristics are summarized in Table 1. Twenty four percent of C − patients had systematic SARS-CoV-2 screening depending on environmental context, 15% had cardiac failure, 20% had respiratory dysfunction and the remaining had diverse disorders (organic dysfunction or other infections). Populations were gender-matched (51% and 49% male in C − and C +, respectively). Age was similar in the groups since mean ± standard deviation (SD) age was 75.9 ± 17.5 in C − patients and 77.5 ± 16.0 years old in C + patients (*p* = 0.83). Importantly, BMI was similar in C − and C + patients (*p* = 0.22). The Charlson comorbidity index as well as distinct known risk factors for severe COVID-19 were similar between the groups (*p* = 0.22). We noted that C + patients had more symptoms as 71% of C + patients had more than 2 symptoms (out of the main 4 symptoms) compared to 48% in C−  patients (*p* = 0.024). Cough was more frequent in C + patients. The delay between plasma sample collection and SARS-CoV-2 RTPCR was similar (2.6 ± 1.8 vs. 3.6 ± 2.6 days in C − and C +, respectively; *p* = 0.07). After correction for multiple testing, biological parameters were not significantly different between groups (supplementary table S1). We observed a trend of lower troponin concentrations for C + patients but it was not significant after correction of Benjamini and Hochberg (raw *p*-value = 0.03, adjusted *p*-value: 0.003).

**Table 1:** Demographical and clinical characteristics of patients infected by SARS-CoV-2 (C +) and controls (C −).

	Non COVID-19 patients (C −)	COVID-19 patients (C +)	*p*-value
Age (years, mean ± SD)	75.9 ± 17.5	77.5 ± 16.0	0.83
Gender (%male)	51.1	49.1	1
Body mass index (kg/m^2^, mean ± SD)	27.9 ± 8.1	25.6 ± 4.4	0.22
Charlson comorbidity index (mean ± SD)	2.9 ± 2.2	2.5 ± 2.3	0.24
**Risk factor**			
Hypertension (%)	56.8	59.2	0.84
Diabete (%)	31.1	25.5	0.65
Dyslipidemia (%)	33.3	20.0	0.17
Cardiovascular event (%)	55.6	50.9	0.69
Smoking (%)	42.3	25.4	0.09
Renal failure (%)	46.7	25.5	0.04
Delay SARS-CoV-2 test-sample collection (days, mean ± SD)	2.6 ± 1.8	3.6 ± 2.6	0.07
**Symptoms at SARS-CoV-2 test time**			
Dyspnea (%)	56.8	64.8	0.53
Cough (%)	38.6	61.1	0.04
Diarrhea (%)	20.5	14.8	0.59
Fever (%)	50.0	66.7	0.10
**WHO ordinal scale (% of patients with scale > 3)**			
D0	43.2	55.6	0.31
D1	32.7	51.9	0.06
D7	15.9	37.3	0.022
**Patients with intubation and mechanical ventilation (%)**			
D0	11.9	17.3	0.56
D1	14.0	17.3	0.78
D7	7.0	20.8	0.08
**Patients' evolution at the 15th day**			
Death (%)	4.5	11.1	< 0.0001
Hospitalization (%)	28.5	66.7	
Hospital discharge (%)	65.9	22.2	

Although the percentage of patients with WHO ordinal scale > 3 was similar between C − and C + groups, it was higher in C + (37.3%) versus C − groups (15.9%) at t2 (*p* = 0.022). The variation of WHO ordinal scale over 7 days showed that 12.7% of C + patients versus 4.5% of C − patients had worse respiratory function (i.e. decrease of WHO scale, NS). Interestingly, the percentage of C − patients under mechanical ventilation decreased from 11.90% at D0 to 6.98% at D7 and it increased from 17.31% at D0 to 20.75% at D7 in C + patients (no significant). Taking into account the specific patients’ management, we found no statistical difference of pO2, spO2, and respiratory frequency at any times between both groups of patients (not shown). Although most of C − patients left the hospital after the acute episode of SARS-CoV-2 suspicion, C + patients were still hospitalized 15 days later, and a higher proportion died (*p* < 0.0001).

### Metabolic findings

According to the process of data pre-treatment, the final dataset contained only metabolites presenting low pre-analytical and analytical variabilities. After filtering regime, we obtained 67 out of 233 metabolites for C18-negative mode, 71 out of 303 metabolites for C18-positive mode and 80 out of 334 for HILIC column. The redundancy was analyzed to keep at the end 160 metabolites. So we kept only 25% of the metabolites initially detected (supplementary table S2). The median CV of the metabolites from the final data set was 10.5%. To have an overview of all metabolic pathways that could be evaluated with reliability in our study, we presented the list of these pathways corresponding to the 160 metabolites retained in the final dataset. Supplementary figure S1.

### Metabolome profile distinguishes C + and C − patients

**Figure 1:**
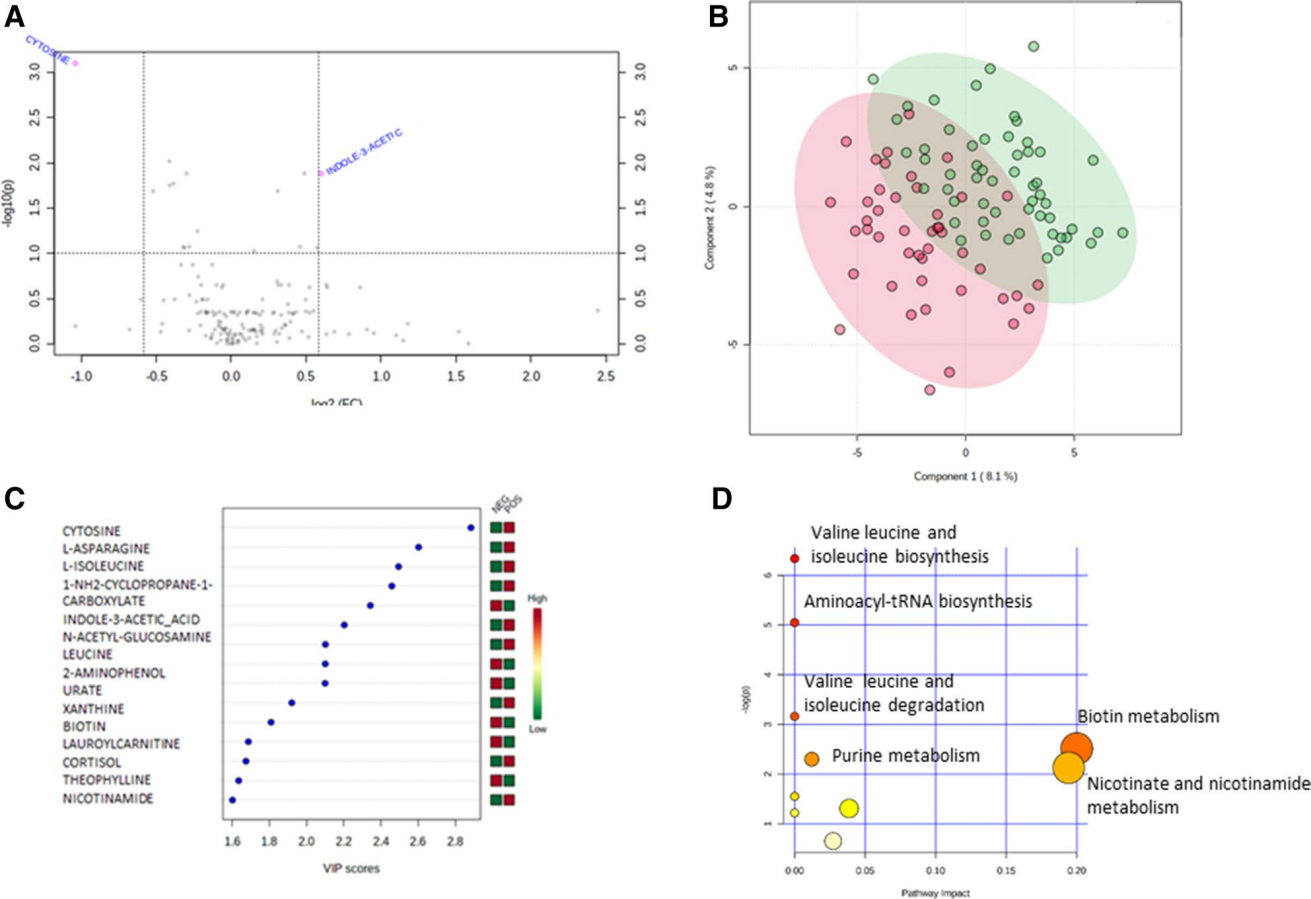
Univariate and multivariate analysis from plasma metabolome profile of C + versus C − patients. (**A**) Univariate analysis via volcano plot based on fold change and adjusted p-value**,** highlighting 2 metabolites, (**B**) Score scatter plot based on the PLS-DA models to explain the diagnosis (pink for C − and green for C +, (**C**) rank of the different metabolites (the top 15) identified by the PLS-DA according to the VIP score on the x-axis. Colored boxes on the right indicate the relative concentrations of the corresponding metabolite in each group, (**D**) pathway analysis based on enrichment analysis procedures, thus identifying the most relevant metabolic pathways via pathway impact and adjusted *p*-value. The figures were drawn via metaboanalyst software v 4.0 (https://www.metaboanalyst.ca/).

Volcano plot analysis (Fig. 1A) highlighted two main metabolites: cytosine (adjusted *p*-value 0.0018, FC = 0.5) and indole-3-acetic acid (adjusted *p*-value: 0.0177, FC = 1.5). Importantly, other metabolites had adjusted *p*-value < 0.05 (but with FC between 0.76 and 1.42) such as 2-aminophenol, L-isoleucine, L-asparagine, 1-NH_2_-cyclopropane-1-carboxylate, L-leucine, urate and xanthine.

Unsupervised analysis (PCA) did not reveal any outsiders. PLS-DA divided patients into C + and C − groups with correct performances defined by an accuracy at 74% and a significant permutation test (*p* < 0.01), ensuring the robustness of the model. The score plot and the important features contributing to this model are represented in Fig. 1B and C, respectively. The eight metabolites that had VIP score higher than 2 were the same as those discriminant between groups after univariate analysis. The metabolic pathways associated with the discriminant metabolites are shown on Fig. 1D and highlighted the metabolism of biotin (only one metabolite), nicotinate and nicotinamide metabolism.

### Metabolome profile can correctly predict SARS-CoV-2 infection on an independent population

**Figure 2:**
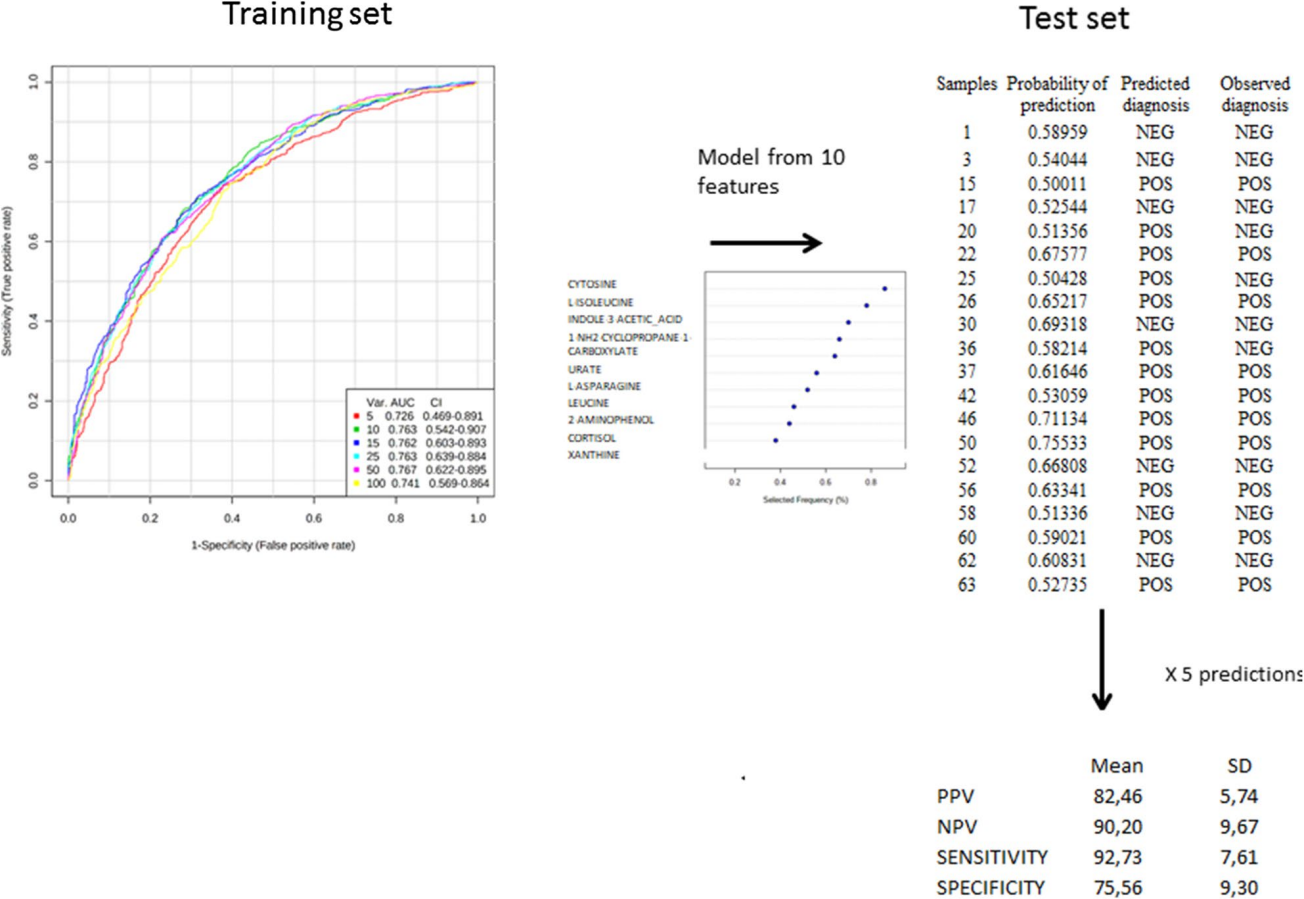
Example of the prediction of SARS-CoV-2 infection in an independent cohort (i.e. test set) from plasma metabolome profile of the patients from the training set. In a training set, ROC curves were obtained after PLS-DA models based on different numbers of metabolites. The model providing the highest Area Under the Curve with less than 10 metabolites was used to predict the diagnosis in the test set. Thus, the ROC curve in the test set enabled to compare the diagnosis prediction to the observed diagnosis. Finally the performances of 5 independent predictions determined by the 4 following performance criteria :sensitivity, specificity, Positive Predictive and Negative Predictive values were calculated. The figures were drawn via metaboanalyst software v 4.0 (https://www.metaboanalyst.ca/).

We obtained quite similar performances of the PLS-DA models in the 5 different training sets (not shown). Figure 2 is based on an example of a prediction (out of the 5 predictions realized) and shows the main results of the different steps previously described to predict the diagnosis in the test set from the training set. The ROC curves built from PLS-DA based on a variable number of features in the training set showed excellent performances. The ROC curve providing the best performances with less than 10 features revealed an AUC at 0.763 for this example of prediction. The 10 features used to predict diagnosis in the test set enabled the construction of a ROC curve with AUC at 0.879 (*p* < 0.01) and provided a probability of group prediction as shown in Fig. 2. In the present case, 3 patients were poorly predicted out of 20. According to the same strategy applied for the 5 different and independent predictions, we obtained satisfactory reproducibility of performance criteria between models with sensitivity, specificity, PPV and NPV > 75% in the independent populations (i.e. test sets).

Interestingly, Venn diagrams (not shown) revealed that 6 metabolites were present in the 5 independent predictions: L-asparagine, 1-NH_2_-cyclopropane-1-carboxylate, cytosine and L-isoleucine, L-leucine and 2-aminophenol. As expected all of them were also discriminant in the entire cohort.

### Metabolome analysis did not predict clinical outcomes of COVID-19 patients with high reliability

**Figure 3:**
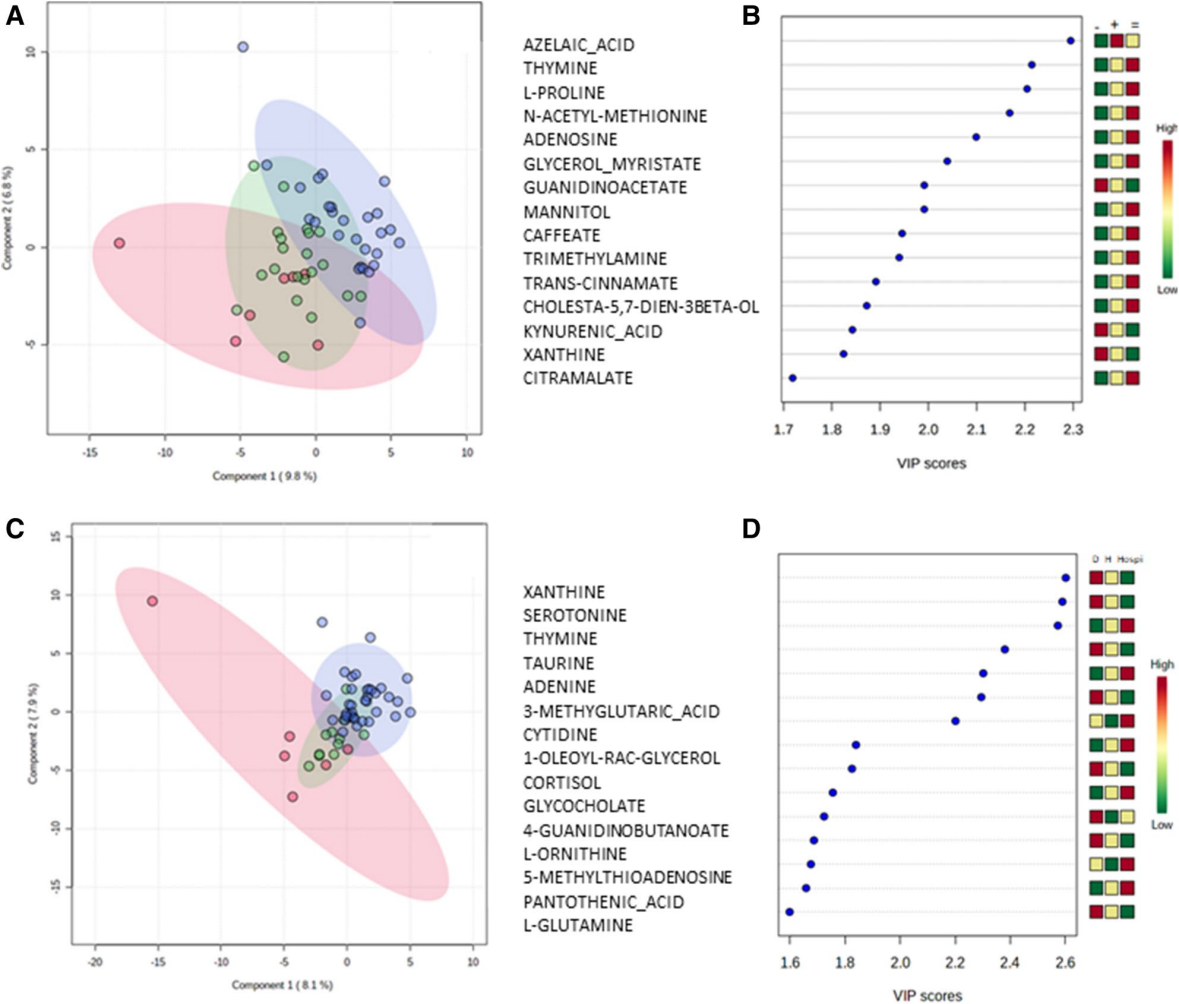
Multivariate analysis from plasma metabolome profile to explain clinical evolution in C + patients. (**A**) Score scatter plot based on the PLS-DA models to explain the evolution of the WHO ordinal scale at D7, red for worst score, green for a better score and purple for a similar score between D0 and D7, (**B**) rank of the different metabolites (the top 15) identified by the PLS-DA according to the VIP score on the x-axis. Colored boxes on the right indicate the relative concentrations of the corresponding metabolite in each group (− , + , = for the evolution of WHO ordinal scale; D: death, H: Home, and Hospi : hospitalization). (**C**) Score scatter plot based on the PLS-DA models to explain the evolution of patients at D15: red for death, green for hospital discharge and purple for hospitalization. Component 1 and 2 represent a linear combination of relevant metabolites expressing the maximum variance. After mean-centering and autoscaling, the data are used for the computation of the first principal component, that is the line in the K-dimensional space that best approximates the data in the least squares sense. Importantly one principal component is insufficient to model the systematic variation of a data set, and a second principal component is calculated. The second PC is also represented by a line in the K-dimensional variable space, which is orthogonal to the first PC. (**D**) rank of the different metabolites (the top 15) identified by the PLS-DA according to the VIP score on the x-axis. Colored boxes on the right indicate the relative concentrations of the corresponding metabolite in each group (− , + , = for the evolution of WHO ordinal scale; D: death, H: Home, and Hospi: hospitalization). The figures were drawn via metaboanalyst software v 4.0 (https://www.metaboanalyst.ca/).

We explored whether metabolome profiles predicted outcomes 7 and 15 days after sampling. After 7 days, the WHO ordinal scale increased (i.e., clinical worsening) in 7 patients, was stable in 27 patients, and decreased (i.e. clinical improved) in 21 patients. Univariate analysis did not highlight any discriminant metabolite. The PLS-DA model showed separation of the subgroups although accuracy was poor (58%, Fig. 3A), with a non-significant permutation test (*p* = 0.65). Among the 15 metabolites involved (Fig. 3B) in the separation of the subgroups based on WHO ordinal scale, we found guanidinoacetate and proline involved in arginine and proline metabolism, as well as xanthine and adenosine involved in purine metabolism. We also tested a model to distinguish only stationary patients from those who improved their respiratory function (i.e. exclusion of patients who became more severe). This model was also not significant.

After 15 days, 6 patients had died, 37 patients were still hospitalized and 12 had been discharged from hospital. Kruskal Wallis analysis of metabolites levels between groups of patients becoming revealed some metabolites presented in figure S2. Multivariate analysis (Fig. 3C,D) showed a model with good accuracy (> 75%) but a no significant permutation test. The model was not improved after exclusion of patients who died. Among the most discriminant metabolites, we found L-ornithine and L-glutamine involved in arginine metabolism, xanthine and adenine involved in purine metabolism, as well as 4-guanidinobutanoate and L-ornithine, both involved in arginine and proline metabolism.

**Figure 4:**
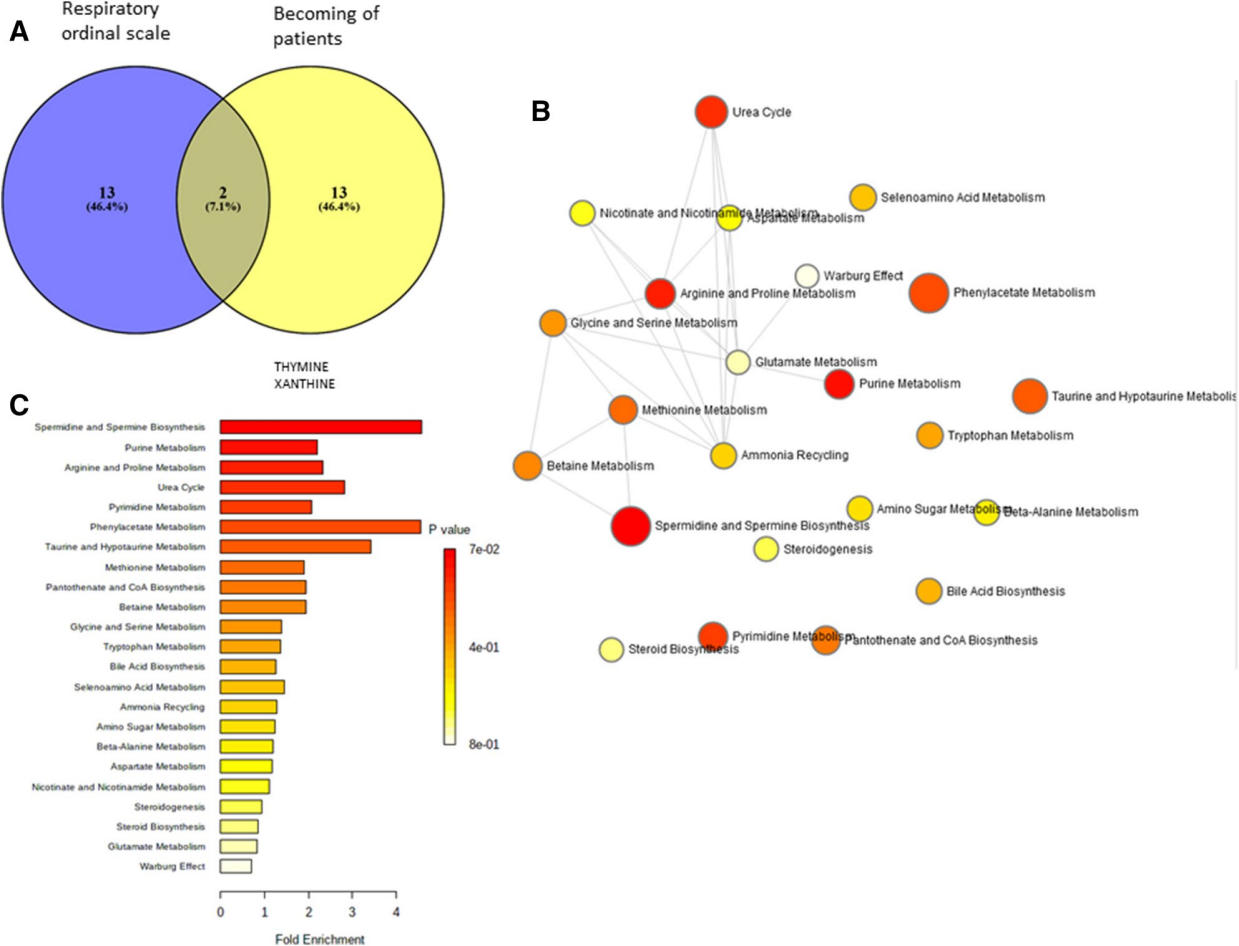
Metabolites the most discriminant for the evaluation of clinical evolution in C + patients, (**A**) Venn diagram (Venny 2.1, https://bioinfogp.cnb.csic.es/tools/venny/) showing the 15 metabolites with the highest VIP used to explain the evolution of WHO ordinal scale (purple) and the evolution of patients (yellow) with the two common metabolites xanthine and thymine; (**B**) enrichment analysis based on the metabolites presented in the Venn diagram (**A**). Each node represents a metabolite set with its color based on its adjusted *p*-value and its size is based on fold enrichment. Two metabolite sets are connected by an edge if the number of their shared metabolites is over 25% of the total number of their combined metabolite sets. (**C**) Metabolites sets overview enrichment. The list of metabolic pathways is summarized under the network view. The enrichment analysis was implemented using the hypergeometric test to evaluate whether a particular metabolite set is represented more than expected by chance within the given compound list. One-tailed *p* values are provided after adjusting for multiple testing. The figures were drawn via metaboanalyst software v 4.0 (https://www.metaboanalyst.ca/).

A Venn diagram including all metabolites found in the 2 models performed to predict clinical outcomes is presented in Fig. 4A. We noted 2 common metabolites, thymine and xanthine and the most involved metabolic pathways associated with clinical outcomes were spermidine/spermine biosynthesis, purine, arginine and proline metabolism (Fig. 4C) that are not directly connected as shared metabolites were < 25% of the total number of their combined metabolite sets (Fig. 4B).

## Discussion

To our knowledge, this study is the first to evaluate the plasma metabolome profile of patients with COVID-19 at the time of the viral diagnosis (very close to their infection, as the screening strategy aimed at the earliest diagnosis) compared to suspected patients for SARS-CoV2 infection with negative RTPCR. Our findings revealed a clear clustering of patients according to the status infected (C +) or not (C −) and also highlighted the power of the multivariate model to predict patients’ diagnosis. The weaker relation between plasma metabolome and clinical severity within the C + group did not allow considering the metabolome profile as a prognostic biomarker, mainly due to the low number of patients with clinical degradation. However, this strategy helped us to identify some interesting metabolic pathways.

### Characteristics of the study population

The population in this study mostly comprised patients with mild or moderate disease both in the C − and C + groups. Criteria for prescription of SARS-CoV-2 RTPCR at our hospital did not change during the recruitment period, ensuring homogeneity of collected data over the inclusion period. Contrary to the recent work studying proteomic and metabolomics profiles of COVID-19 patients^11^, our samples were collected at the time of the diagnosis in a population naïve for anti-viral treatment. Taken into account the heterogeneity of disease evolution, this difference of inclusion criteria may explain some different findings. Although the C + and C − populations were comparable for most parameters, there was a higher frequency of cough and a higher proportion of patients with more than 2 symptoms in the C + group. Both C − and C + were frequently overweight^16^. Importantly, age and BMI were similar between groups. Likewise, diabetes was as frequent in both groups, thus limiting the putative repercussions of differential complications of diabetes on metabolome pattern^17–19^. As metabolites concentrations may be associated with BMI, diabetes or age for example, these parameters must be carefully controlled as it was done in this present study. Altogether, the matching of both groups suggested the absence of confusion factors that could alter metabolomics findings in our study.

A fraction of patients with COVID-19 experiences clinical worsening typically 7–10 days after diagnosis^20^, thus we collected clinical data 7 and 15 days after SARS-CoV-2 RTPCR to assess ability of metabolome profiles to predict outcomes. The fraction of patients under mechanical ventilation among C + patient at D7 (20.75%) contributes to the increased WHO ordinal scale, linked with respiratory failure described in the literature^21^. As expected, the decrease of the WHO ordinal scale from D0 to D7 was less frequent in C + patients. Likewise, patients of the C + group were more likely to be hospitalized or deceased at the 15th day after diagnosis, comparable with literature data^5^. This probably directly reflected severity of COVID-19 rather than other ailments since both groups were otherwise similar at baseline. In addition, since our hospital was not saturated even at the peak of the COVID-19 outbreak, it is unlikely that unusually early discharge of C−  patients accounts for the difference in hospitalization rates between groups.

### Correct prediction of SARS-CoV-2 infection from plasma metabolome profile

This study relied on metabolomics analysis of plasma samples. Although analysis of respiratory samples might allow to directly address the mechanisms driving COVID-19 pneumonia, which is the main driver of mortality, plasma is a relevant fluid for metabolomics analyses because: (1) COVID-19 disease involves multiples organ systems beyond the respiratory tract including liver, kidney and the immune system and (2) SARS-CoV-2 virus is rarely present in blood, in contrast with respiratory samples, in which high titers can be detected, but systemic metabolic consequences are expected^22^.

The strategy used in the present study overcomes many of the obstacles of metabolomics experiments through robust internal validation coupled with a validation in an independent cohort (performed 5 times, randomly). The step of independent validation adds robustness to the important concepts of reproducibility, validity, and generalizability, and produces independent confirmation of metabolic markers^23^. In this context of not overfitting modelling, we found correct performances of prediction (criteria > 75%), that would lead us to pursue, and to combine metabolome profile to other parameters, such as inflammatory factors for example. We highlighted some highly discriminant metabolites (cytosine, indole 3 acetic acid) and the pathways of biotine and nicotinate, nicotinamide metabolism.

### Involvement of tryptophan-nicotinamide pathway in SARS-COV-2

Univariate and multivariate analyses have identified two metabolites that are central in the tryptophan-nicotinamide pathway. The 3-indole acetic acid is a breakdown product of tryptophan metabolism. Nicotimamide, an amide derivative of nicotinic acid, is a precursor for generation of the coenzymes NAD + and NADP +, which are essential for many metabolic pathways. The tryptophan-nicotinamide pathway consists of two parts. The first part is from tryptophan to quinolinic acid, and the second is from quinolinic acid to N1-methyl-2-pridone-5-carboxamide and N1-methyl-4-pridone-3-carboxamide including the NAD cycle and nicotinamide catabolism^24^. These metabolomics findings may be linked with the largely described inflammatory signals of tryptophan-kynurenine metabolism^25,26^. The role of peptidyl-dipeptidase A 2 (ACE2) has been largely described in SARS-CoV-2 infection^27^. ACE2-dependent changes in epithelial immunity and the gut microbiota can be directly regulated by tryptophan^28^. The tryptophan-nicotinamide pathway can also act on mTOR activation, which is involved in cell proliferation, survival, transcription and expression of intestinal antimicrobial peptides^29^. The putative consequences of antimicrobial peptides on the intestinal composition of the gut microbiota open perspectives of microbiome role in this infection^30^. Interestingly tryptophan represents a metabolic node that involves serotonine synthesis, kynurenine pathway and the indole/aryl hydrocarbon receptor (AHR) pathway. Indole acetic acid is a ligand of AHR that has been involved in many diseases involving immune and inflammatory processes. All these mechanisms also found in microbiota support the promising perspective of combining peripheral metabolism and microbiome exploration^31^. Omics findings of Shen et al.^11^, and more recently Thomas et al.^32^, also reported the activation of kynurenine pathway in COVID-19 patients. They suggested that NAD synthetized from tryptophan modulates macrophage activity such as the release of interleukin-6 and tumor necrosis factor alpha^33^. Moreover Farsalinos et al*.*^34^ considered SARS-CoV-2 as a disease for the nicotonic cholinergic system. They suggested that the inflammatory response observed in SARS-CoV-2 patients^7,35^ leads to clinical characteristics that could be linked to a modification of the cholinergic anti-inflammatory pathway. Consequently, this group suggested that nicotine should be protective in SARS-CoV-2 patients due to its anti-inflammatory role. Metabolomics applied to therapeutics i.e. pharmacometabolomics should be of great interest in this context.

### Increased cytosine levels in COVID-19 patients

Cytosine was the main discriminant metabolite between C + and C−  patients. Cytosine belongs to the pyrimidine class and is one of the four main bases found in DNA and RNA. Viral infections are known to cause significant metabolic changes in host cells, such as upregulation of pyrimidine nucleotide biosynthesis. Danchin A et al.^36^, reported the importance of cytosine as a coordinator of cell metabolism in SARS-CoV-2. It has been previously shown that the base composition of human mRNA and SARS-CoV-2 RNA is quite different. Indeed, the cytosine amount is lower in SARS-CoV-2 RNA genome (17.6% C vs. 30.2% A, 19.9% G, 32.4% U) than in human RNA. The increased plasma cytosine levels in COVID-19 patients may correspond to the coupling between synthesis of viral particles and the host cell’s metabolism and this loss in cytosine may be associated with escape innate immunity. Moreover, cytosine appears as key in the virus evolution as cytosine availability drives RNA virus evolve a new progeny^36^. According to our findings, we suggest that the reduced percentage in cytosine in the SARS-CoV-2 genome may be associated with increased levels in biological fluids of the host, as the synthesis machinery of bases may be increased, cytosine is probably few used by this virus and cytosine may be released by cell lysis inherent to infection.

### Modest association between metabolic disturbance and COVID-19 severity

The impact of the immune response on SARS-CoV-2 severity is still an enigma and we suggested that metabolic status may be an additional tool to characterize clinical evolution. The most described predictors of disease severity are older age and comorbidities^4^. PLS-DA models (Fig. 3) showed a mild separation between groups but with a high recovery between these groups, and a high heterogeneity within each subgroup of disease evolution. Although exclusion of the most severe patients showed a better separation of groups, this was most probably due to the low statistical power which was more appropriate to separate 2 instead of 3 groups in small population. Other types of modelisations have been performed (Othogonal PLS-DA, random forest), but the models were still not significant (not shown).

Purine and pyrimidine pathways [i.e. xanthine, thymine (Fig. 4A)] have been highlighted and may be linked with purine and pyrimidine release from cell lysis^37^. ATP and NAD^+^ are described as excitatory molecules and adenosine as anti-inflammatory effector on immune cells. Importantly, purines play a crucial role in controlling the activation and differentiation of immune cells. Inflammation induced by SARS-CoV-2 infection may be controlled in part by the release and metabolism of purines, modulated by environmental factors such as hypoxia^37^. As these findings are consistent with the involvement of tryptophan-nicotinamide pathway previously discussed, further studies with a complete evaluation of inflammation status and evolution would be of great interest to better understand both mechanisms in this infection. Consistently, the relation between metabolomics profile and inflammatory cytokines evaluated in mice with H1N1 influenza virus infection showed effects of the virus infection on tryptophan and other amino acids, and effects on pathways such as purines, pyrimidines and lipids^38^. Moreover, other mechanisms may be related to purine and pyrimidine release independently from cell lysis^39^, for example in response to biochemical or mechanical or physical stimuli. The nucleotide storage and release from secretory granules, described in airway epithelial ciliated and goblet cells in lung tissues may be involved in such kind of infection and may explained our findings^39^.

Arginine and proline metabolism as well as end products (polyamine: spermidine) were also related with clinical evolution. Arginine is an essential aminoacids for NO homeostasis and polyamines are small aliphatic polycations, ubiquitously found in living cells that have multiple functions, which are only partially understood^40^, including protection against stress induced by Reactive Oxygen Species (ROS)^41^ and the induction of autophagy^42^. Interestingly our findings are consistent with those of Shen et al.^11^, who described enrichment in some of amino-acids, including metabolites involved in arginine metabolism.

## Perspectives and conclusion

To our knowledge, this work represents the first metabolomics study into early diagnosis or prognosis biomarkers of COVID-19. Whether metabolomics analysis can provide such a tool remains to be explored in prospective studies, but to date, we report a significant plasma metabolome profile of COVID-19 patients with involvement of the tryptophan-nicotinamide pathway as well as cytosine metabolism. This strategy has to be considered as helpful to characterize patients, to determine homogeneous subgroups of infected patients, probably in combination with viral biomarkers. Metabolomics must be applied on an independent cohort with more severe patients and in parallel to inflammation exploration to confirm the suspected mechanisms associated with this infection. Omics approaches in host cells infected by this virus may complete the data about the dynamics of metabolism-inflammation link, as well as the short and long term consequences on cells homeostasis. Our findings open the perspective of omics combination with proteomics and lipidomics to expand the metabolic coverage^43^.

## Supplementary information

Supplementary Figure 1.

Supplementary Figure 2.

Supplementary Table 1.

Supplementary Table 2.
