# Supplementary figures and images for: The specific metabolome profiling of patients infected by SARS-COV-2 supports the key role of tryptophan-nicotinamide pathway and cytosine metabolism
Source: Sci Rep. 2020 Oct 8;10:16824. doi: 10.1038/s41598-020-73966-5 (PMC7544910; doi:10.1038/s41598-020-73966-5)

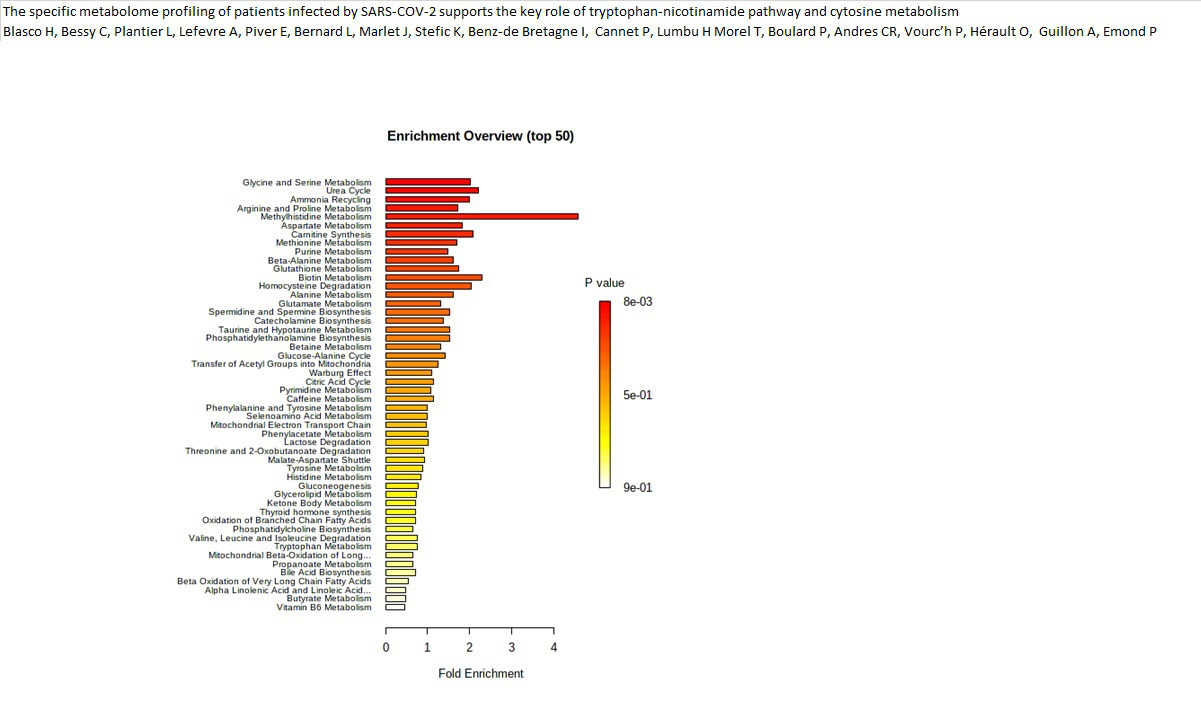

Supplement: Supplementary file 1 — Supplementary Figure 1. [file 41598_2020_73966_MOESM1_ESM.jpg]

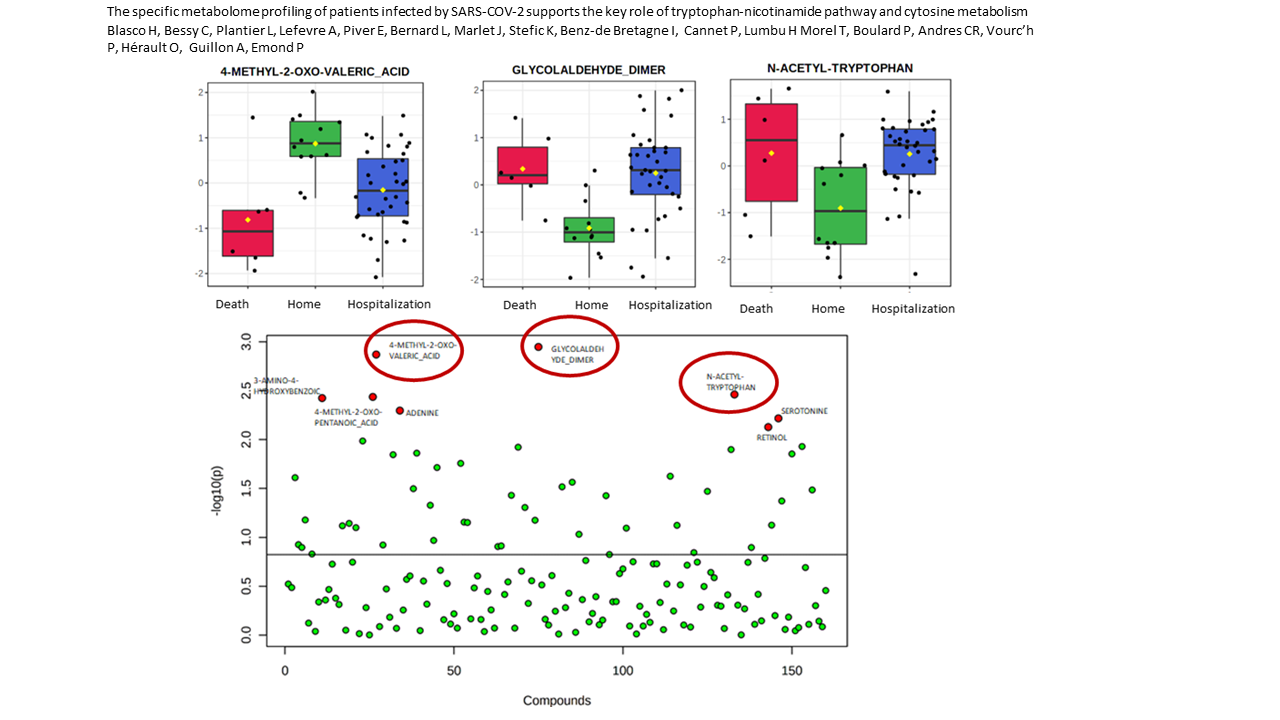

Supplement: Supplementary file 2 — Supplementary Figure 2. [file 41598_2020_73966_MOESM2_ESM.tif]
